# Potential Effects of Corneal Cross-Linking upon the Limbus 

**DOI:** 10.1155/2016/5062064

**Published:** 2016-09-05

**Authors:** Johnny E. Moore, Davide Schiroli, C. B. Tara Moore

**Affiliations:** ^1^School of Biomedical Sciences, University of Ulster, Coleraine BT52 1SA, UK; ^2^Cathedral Eye Clinic, Academy Street, Belfast BT15 1ED, UK

## Abstract

Corneal cross-linking is nowadays the most used strategy for the treatment of keratoconus and recently it has been exploited for an increasing number of different corneal pathologies, from other ectatic disorders to keratitis. The safety of this technique has been widely assessed, but clinical complications still occur. The potential effects of cross-linking treatment upon the limbus are incompletely understood; it is important therefore to investigate the effect of UV exposure upon the limbal niche, particularly as UV is known to be mutagenic to cellular DNA and the limbus is where ocular surface tumors can develop. The risk of early induction of ocular surface cancer is undoubtedly rare and has to date not been published other than in one case after cross-linking. Nevertheless it is important to further assess, understand, and reduce where possible any potential risk. The aim of this review is to summarize all the reported cases of a pathological consequence for the limbal cells, possibly induced by cross-linking UV exposure, the studies done* in vitro* or* ex vivo*, the theoretical bases for the risks due to UV exposure, and which aspects of the clinical treatment may produce higher risk, along with what possible mechanisms could be utilized to protect the limbus and the delicate stem cells present within it.

## 1. Clinical Applications of CXL

In the last decade corneal cross-linking (CXL) has become the routine treatment for progressive ectasias including keratoconus (KC) and Pellucid Marginal Degeneration (PMD) [1, 2]. This approach exploits the combined properties of ultraviolet A (UVA, 315–400 nm) and riboflavin: UV irradiation excites the fluorescent molecule to a triplet state, with consequent generation of a singlet oxygen and superoxide radical. These radical products are then able to strengthen the corneal stroma and also possibly more importantly increase the stromal resistance to enzymatic degeneration [3] forming covalent bonds in the collagen.

This process is also cytotoxic, as planar molecule riboflavin intercalates between the bases of DNA and RNA and, once activated, it is able to oxidate the nucleic acids [4–6]. Thanks to this characteristic CXL has been used in transfusion medicine to diminish the risk of transfer of infectious agents [7] and more recently has become a recognized technique as a possible adjunctive or primary treatment for infectious keratitis [8, 9]. Moreover cross-linking is able not only to kill infective organisms within the corneal stroma but also to arrest the corneal melting process caused by the release of proteolytic enzymes by both microbes and invading protective white blood cells [10–12].

## 2. Commercial CXL

Several alternative methods to perform CXL have been developed as summarized in various literature reviews [13]. The advantages and disadvantages of the different techniques in terms of the limbal safety are discussed later in this review.

### 2.1. Standard Epithelium-Off CXL

In the standard CXL the central part of the cornea (8-9 mm) of the cornea is irradiated with UVA at 3 mW/cm^2^ for 30 min. The corneal thickness has to be checked in the pre-CXL procedure and has to be greater than 400 *μ*m, to avoid damage to the endothelium. To enable the complete penetration of the riboflavin in the stroma the epithelium is debrided. Topical antibiotics and corticosteroids are prescribed after the procedure until corneal reepithelialisation.

### 2.2. Accelerated CXL

Accelerated CXL protocols exploit UVA energies with higher fluencies and shorter exposure times. In this way, following the Bunsen-Roscoe law of reciprocity, the endothelial UVA dosage can be kept constant, below the cytotoxic threshold, but with the same efficiency. This is potentially an advantage for patient safety, as the time in which the keratocytes are exposed to UVA is reduced, with probably a decreased rate of damage and apoptosis. Some clinical studies have demonstrated the effectiveness of this technique and more studies are currently ongoing.

### 2.3. Epithelium-On (Epi-On) CXL

Epithelial debridement is necessary to allow stromal diffusion of riboflavin: as confirmed by several studies riboflavin hydrophilic nature stops it from penetrating the tight junctions of the intact epithelial barrier.

The development of an epi-on CXL is however desirable to reduce risks of keratitis and of other possible complications. For this reason several alternative solutions have been developed; between them the most ones promising are some novel formulations of riboflavin, which facilitate the transepithelial absorption, and iontophoresis [13, 29]. This last option in particular is giving encouraging results also in clinical studies, as it is discussed in the epi-on chapter. The nature of the small riboflavin molecule which is negatively charged at physiological pH and soluble in water makes it highly suitable for iontophoretic transfer.

## 3. Clinical Complications of CXL

The results from an increasing number of long-term studies have recurrently demonstrated that this is a safe method but there are also various different complications observed after CXL treatments [30, 31], as summarized in Table 1. One major problem is the increased risk of infective keratitis due to delayed reepithelialisation, [14, 15, 18, 32, 33] along also with cases of sterile peripheral corneal infiltrates [34]. The incidence of infective keratitis, as indicated from these published cases, would appear to be significantly higher than that reported in a very similar procedure called photorefractive keratectomy (PRK) [35]. IN PRK an identical 9 mm diameter epithelial defect is created in the cornea prior to treatment of the corneal stroma with a short UV wavelength 193 nm excimer laser, while in CXL a longer UVA wavelength (360 nm) is utilized for a much longer period of time.

In response to the increase in infective keratitis, which may result from a localized alteration in corneal immune status, clinicians have modified their postoperative treatment advice often dispensing with the use of CLenses and increasing the frequency of antibiotic usage (unpublished data).

Epithelial-on (epi-on) CXL further decreases the possibility of contracting keratitis, as in this case the important epithelial barrier is kept intact [2]. Recent advances in the epi-on CXL, like iontophoresis and transepithelial CXL [36, 37], improve the transfer of riboflavin facilitating deep stromal penetration, making the epi-on CXL a potentially safer alternative to the standard epi-off CXL with comparable clinical outcomes.

In parallel with the risk of keratitis a major concern is the possibility of inducing toxicity or cell death to the endothelium, keratocyte, and limbal cells. The risk of damaging the endothelium appears to be minimal if certain stromal thickness levels are maintained prior to treatment. Oxygen free radicals and superoxide radicals, however, cause significant keratocyte toxicity and death [17, 38]. This cellular toxicity is however limited to the anterior 300 *μ*m with a toxic cellular threshold of 0.5 mW/cm^2^ for 30 mins of treatment. Possible damage to the endothelium could be a problem as it lacks regenerative capacity, but cell density, morphology, and cell count were demonstrated to be unaltered as long as the criteria of maintaining 400 *μ*m of minimum stromal depth, which ensured sufficient absorption of UVA exposure to prevent attainment of the toxic threshold of 0.35 mW/cm^2^ from a 30 min exposure [3]. Moreover riboflavin itself has the role of photosensitizer but it also absorbs the UVA radiation furtherly protecting the endothelium, so for a thin stroma it might be possible to increase the amount of riboflavin for improving the UVA protection through the stroma [29].

An interesting matter of debate is instead the toxic threshold for possible UVA/riboflavin induced oxidative damage to the permanent epithelial and/or anterior stromal stem cells of the eye contained in the limbal niche, about which there are only a few reported studies.

## 4. UV-Induced Mechanisms of Damage

UVA has effect on various cellular chromophores, like flavins and amino acids (e.g., tryptophan, tyrosine, and histidine). Reactive oxygen species (ROS: superoxide anion O_2_^•−^ and the hydroxyl radical OH^•^), as well as nonradicals like hydrogen peroxide (H_2_O_2_ and ^1^O_2_) are then generated by these molecules after the UV absorption.

Mammalian cells have developed two main systems to protect themselves from the ROS oxidative stress, which represents the major cause of risk and the initial step for the developing of an UV-induced skin cancer.

The first mechanism of protection is the nonenzymatic antioxidants, *α*-tocopherol, ascorbic acid, glutathione, and *β*-carotenoids, while the other one is constituted by the enzymatic antioxidants such as superoxide dismutase (SOD), catalase, and glutathione peroxidase (GPx) [39].

UVA irradiation induces cyclobutane pyrimidine dimers (CPDs) in DNA, while both UVA and UVB can promote the formation of oxidized DNA, like 8-oxo-7,8-dihydro-2′-deoxyguanosine (8-oxo-dG).

This is the most frequent UVA-induced oxidative base lesion and it can cause the G to T transversions. 57% of the mutations occurring after UVA treatment have been reported in fact at the TT sites (with C, CT, or CC sites at 18, 11, and 14%, resp.) [40].

In human squamous cell tumors the G-T transversions are more common than the C-T, showing a specific fingerprint mutations that strongly associate the UVA-induced DNA damage to human skin carcinogenesis [41].

Several* in vitro* experiments have been reported to characterize the amount and the spectra of the possible lesion and mutation, but these are quite variable, depending on the actual experimental conditions, and, due to the low mutagenic potential of UVA, it is quite difficult to quantify the ratio between the mutation induction and the UV dosage [42].

Despite these difficulties most of the studies seem to be in accordance with the fact that UVA induces a higher number of delayed mutations with respect to UVB and X-radiation although only few immediate mutations are produced [43–46].

## 5. Consequences of UV Exposure on Ocular Surface and Limbus

### 5.1. UV Ocular Surface Exposure Diseases

A wide range of different pathologies have been associated with UV exposure and they can affect different parts of the eye, including cataract and retinal macular degeneration. UV exposure has been further implicated in several diseases involving the conjunctiva and cornea like pterygium and pinguecula, photokeratitis, keratopathy, and ocular surface squamous neoplasia [47].

### 5.2. UV Exposure and Eye Cancer

It is well accepted that UV plays a major role as a mutagen in different pathologies, firstly cutaneous cancer [42]. It is similarly well established that the occurrence of cancers is related to sun exposure and hence skin cancers are more common in nontanning individuals, areas of the body with the highest sun exposure (face, ears, and backs of hands), and regions with high levels of UV exposure [48].

Ocular surface cancers are quite rare in the general population, testifying to the ability of the innate system to manage UV-induced cellular changes on this surface, but it is also true that the incidence is much higher in countries such as Australia, where there are high numbers of Caucasians. These lesions seem to focus anatomically around the limbal region in keeping with the presence of long-lived stem cells in that region.

Usually in short-lived cells a mutation does not tend to represent a problem because it disappears with cell death, but it can represent a serious problem in long-lived cell such as the limbal stem cells. They potentially survive for the whole life of the individual and hence the propensity to accumulate oncogenic damage over time makes it more likely to result in invasive cancer. As the epithelial stem cells in the cornea are specifically retained at the peripheral limbal region this is in keeping with the high incidence of ocular surface cancer found to be present in this region [48].

### 5.3. The Role of the Limbus

The limbal region, situated at the anterior portion of the cornea, hosts the stem cells involved in the corneal epithelium turnover. Their role in maintaining the health of the corneal epithelium over a lifetime is fundamental for the correct functioning of the cornea and deficiency or loss of these cells is associated with a characteristic phenotype of the ocular surface consisting of an irregular epithelium, with conjunctival epithelial ingrowth, vascularization, goblet cells, recurrent epithelial breakdown, and chronic surface inflammation [49, 50].

These limbal stem cells are attached to the basement membrane and deep within the valleys of an undulated region of stroma called the palisades of Vogt [51, 52]. They are usually maximally concentrated in the superior region of the limbus normally protected by the upper lid and in the inferior limbus, the area protected by the lower lid [53]. The vascularization and pigmentation of this area are thought to take also part in the physical defense of the stem cells from UV exposure [54, 55]. Similarly to what happens in the skin sporadic melanocytes were in fact founded in the palisades of Vogt, they have dendritic processes surrounding the basal limbus epithelial cells expressing K19 (+), and they form a melanin unit that protects the limbus from the UV. Melanin has in fact antioxidative properties and it might hence protect from the UV-induced oxidant formation in the cornea epithelium [56].

**Figure 1:**
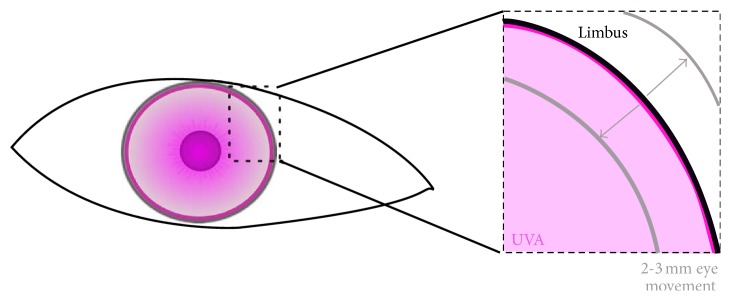
Scheme of the eye treated with CXL: the pink area represents the UVA treated region, while the black line represents the limbus. Small movements of the eye (2-3 mm) can cause the shift of the limbal area into the unsafe region underlying the UVA beam (8 mm diameter).

**Figure 2:**
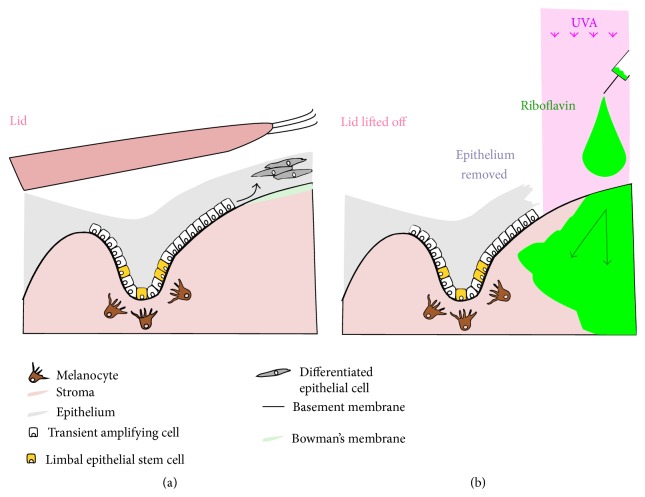
Scheme representing the limbus region untreated (a) and treated with CXL (b). In (b) the lids (superior and inferior) are lifted off and the epithelium is removed. In this case limbus might be only partially protected by the overlaying epithelium. In fact, even if the remotion is accurate, part of the limbus-protecting epithelium could be detached and lose its shielding properties. Moreover the reactive riboflavin is free to diffuse and to reach also the limbal region.

Moreover recent studies have also defined a stromal keratocyte stem cell pool within the anterior stroma also underneath and adjacent to the epithelial palisades of Vogt [48] (Figures 1 and 2).

## 6. CXL: UV Damage of the Limbal Cells

### 6.1. The UV Damage of the Limbus after CXL Treatment

As outlined earlier, CXL induces cytotoxicity and keratocyte cell death [57–59], but generally this does not seem to affect the subsequent clinical epithelial surface once reepithelialisation has occurred.

During the process of clinical CXL the superior and inferior limbal region, which are shown to have maximum stem cells and which are normally hidden by the upper and lower lids [53], are now no longer protected from the prolonged iatrogenic UVA exposure. It is a worry to clinicians as to whether mutagenic changes could be induced within the corneal limbal stem cells during this treatment and any ensuing problems may not show themselves until much later in life.

Though during CXL the limbus of the eye is not deliberately treated, however, it is very difficult, without using a regional anesthetic block to cause extraocular muscle paresis, to adequately protect the limbus from UV exposure during the procedure (Figure 1).

This risk is also higher in the cases of treatment of pellucid marginal corneal degeneration (PMD), where the irradiated area is often peripheral and close to the limbus [60]. The removal of the central epithelium increases the amount of riboflavin transferred into the peripheral cornea and limbal region, greatly enhancing the oxidative effect upon cells affected by UVA within that region. During the deepithelialisation further changes, such as a slough of some of the overlying layers, actually at the limbal region, of epithelium can occur. These layers normally absorb 20% of the UVA passing through the cornea [29, 61]. This will again remove some further aspects of the normal protective anatomical barriers from UVA damage we previously outlined regarding the position of the corneal stem cells located in two niches (the palisades of Vogt and the epithelial crypts). Melanin within the basal region of the limbal epithelia normally acts as a further shield and protector against irradiation. This is supported by the fact that Wollensak and collaborators found viable keratocytes in the deeper layers of the cornea after riboflavin-UVA [62]. However all this resident protection which functions exceptionally well in normal life may not be sufficient to adequately guarantee the safety of the limbal niche within the altered clinical situation of corneal CXL [63].

Several studies have now focused upon this issue, suggesting the potential damaging effect of CXL treatment upon the limbus with the consequent risk of subsequent morbidity for the patients, particularly of developing ocular surface cancer, later in life.

Many publications report studies which demonstrate the risk of potential iatrogenic limbal damage.

The expression of proapoptotic genes was shown to be induced by CXL in an* in vitro* study [63]. Moreover CXL seems to inhibit the regeneration of cultured human limbal epithelial cells [62] and of cells extracted from cadaver eyes previously treated with CXL [64].

*Ex vivo *(corneas from donor) analysis confirm these results, showing the UV damage to the limbal epithelial cells through the measurement of DNA damage markers and oxidative damage of nuclear DNA [65], while in a recent case study a patient treated by CXL has developed a conjunctival intraepithelial neoplasia (the preliminary stage of invasive squamous cell carcinoma) [66]. This last publication represents to date the only* in vivo* reported case demonstrating such a deleterious effect of CXL upon the limbus as the other* in vivo* study [38], done on rabbit eyes, did not demonstrate a pathological effect upon the limbus.

A study on the incidence of the AA-TT mutation caused by the UVA exposure mutations on the DNA of treated cells or tissues might be useful to further confirm the damaging role of the UVA on the limbal cells, as described for the UVA fingerprints analysis in skin cancer [67].

Given the significant potential risk of damage to the limbus it is advisable to exclude from CXL treatment any subjects where there is further comorbidity which could increase the likelihood of induced limbal stem cell problems. This is the case where there is preexisting underlying limbal stem cell deficiency (LSCD) or xeroderma pigmentosum.

## 7. How to Reduce the Limbus Exposure

The before mentioned issues raise several questions: are there better ways by which the limbal region containing stem cells could be protected during the CXL procedure? Could CXL particularly for the limbal region be achieved in any other nontoxic fashion without the use of UVA?

### 7.1. Physical Protection

Usage of polymethyl methacrylate rings, Vidaurri rings, or other rings with UV blockers can prevent CXL-induced limbal stem cells damage [38]. These physical blocking methods seem to provide only partial protection of the limbus with only 20% of the epithelial stem cells coming from treated cadaver that maintains the stemness [68]; one of the main problems in effective protection using this methodology is the constant ocular movement. Other researchers recently proposed the utilization of 8.5 mm punched UV-block contact lenses during CXL [69].

### 7.2. Accelerated Corneal Cross-Linking

A recent study that compared the accelerated corneal collagen cross-linking (ACXL) with the normal CXL on* ex vivo*-cultured limbal epithelial cells proved that the first one is safe [70–72]. This procedure utilizes an increased intensity of UV exposure in parallel with a decreased duration (in the standard CXL the intensity is 3 mW/cm^2^ and the duration is 30 min, while in ACXL these parameters can vary between 30 mW/cm^2^ for 3 min, 18 mW/cm^2^ for 5 min, and 9 mW/cm^2^ for 10 min. More recently an ultrafast version has been released to the market providing an irradiance intensity of 43 mW/cm^2^ [36]) with the total amount of delivered energy that does not change between the two treatments [73–75]. The effective ability of the accelerated CXL in producing the same results to classical CXL is still a matter of debate, with several contrasting results [76]; however quite recently Cheng and collaborators showed how the conventional method still seems to have a better clinical outcome, with greater corneal flattening and reduction in mean keratometry. The demarcation line was also shown to be deeper in conventional CXL [77].

This comparison demonstrates that the accelerated technique still needs further improvement prior to acceptance within the clinic. Moreover, despite the fact that several comparative clinical studies have been conducted [2], there is not as yet an accepted unique protocol for ACXL and large clinical trials are needed.

### 7.3. Chemically Accelerated Cross-Linking

CXL might be accelerated using different compounds alternative to riboflavin or along with it. Vitamin-E was for example proven to enhance the riboflavin solution, shortening the time of exposure, as confirmed in a clinical study with 19 patients. In this case the UVA was irradiated for 2 times, 5 min. at 3 mW/cm^2^ each, with final results comparable to the one of the canonical CXL [78, 79].

### 7.4. UV Devices

It is possible to direct the treatment in a delimited area using some of the specific devices for UV irradiation. An area of 8 mm in diameter, which can be selected in all the modern CXL devices (Table 2), should avoid UV to the limbus, the sclera, or the goblet cells; however even using an 8 mm irradiating device, eye movement makes it almost impossible to avoid limbal irradiation. One of the current modern devices called UV-X*™* utilizes a special radiation homogenizer, to prevent endothelial damage by preventing local radiation spikes [17]. More studies are necessary however to confirm that this treatment is beneficial in also preventing damage to the limbus and particularly the limbal basal epithelial cells.

### 7.5. Antioxidant Treatment

UVA-induced mutations have been demonstrated to occur long after the initial exposure [43]. Various intracellular enzymes are induced in response to UVA light induced oxidative stress and reactive oxygen species (ROS); these include superoxide dismutase (SOD), catalase, and glutathione peroxidase (GPx) [39]. This has led to various attempts to counteract oxidative damage through the use of nonenzymatic antioxidants [80].

During CXl however, it would be counterproductive to try to utilize such methodology, as this could block the actual desired effect of the treatment which utilizes the oxidative stress to induce cross-linking and therapeutic effect. A potential way to protect the limbal stem cells while still achieving the desired oxidative induced effect would be to assess the possibility of upregulation of an intracellular protective response against ROS. Examples of this type of approach might include single or repetitive very low-dose UVA to attempt to upregulate the various antioxidant intracellular enzymes such as manganese superoxide dismutase (MnSOD) or glutathione peroxidase (GPx) [39, 81]. Similar strategies have been used in other forms of medicine using ischaemic reperfusion models where pretreatment with low-dose recurrent ischaemia protects against subsequent oxidative damage [82].

### 7.6. Epithelium-On (Epi-On) Cross-Linking

CXL can be performed while avoiding removal of the epithelium; several studies and reviews analyze this interesting new area of research. The main techniques use either epithelial tight junction disruptors or iontophoresis [76, 83]. Iontophoresis (I-CXL) in particular seems to be the most promising technique to enhance the delivery of riboflavin in an epi-on CXL, even if still not providing the same level of efficacy as classical CXL is currently the most promising transepithelial technique [29, 36]: The riboflavin penetration into the corneal stroma as well as the corneal rigidity was found* ex vivo* to be comparable to that obtained with standard CXL [37, 84]. Moreover another study showed low toxicity from I-CXL, even if the results of the concentration of riboflavin in the corneal stroma in this case were less as demonstrated by HPLC, found 2-fold less concentrated with respect to standard epi-on CXL [85]. However the mechanical strength of the cornea measured in the transepithelial I-CXL was shown to be comparable to the one measured with the standard procedure [86]. Clinical results published for I-CXL with a short 15-month followup show I-CXL to be equally effective at stopping the progression of keratoconus and improving keratometric and visual parameters. long-term clinical outcomes however have still not been investigated [87–90]. Various other epithelium-on techniques, which exploit different formulations of riboflavin with an increased stromal absorbance, show inconsistent results: in a randomized clinical trial with one of the commercial protocols showing keratoconus still progressing 1 year after treatment in 23% of the cases [91]; and this seemed to be confirmed by* in vitro* studies on the available commercial protocols [87] and by other preliminary clinical studies [87]. Opposite results however have been reported by a couple of other clinical studies [92, 93], with positive outcomes demonstrated up to 18 months after the treatment. Further clinical and* in vitro* studies are therefore necessary to assess the status of the current available protocols while, in parallel, it is desirable that research continues upon novel formulations able to increase the bioavailability of riboflavin within the stroma. It is known that the epithelium absorbs about 20% of UVA radiation; therefore clinicians and researchers need to take this into account if they want to deliver enough energy to the stroma while keeping this below the endothelial toxicity threshold [29].

Despite the research that still needs to be done, epi-on procedures are very promising, as they can be applied to patients with a corneal thickness below 400 *μ*m [94]; they can also reduce postoperative pain and similarly the risk of infective keratitis and improve the healing time.

## 8. Future Research: Alternatives to CXL

Many alternative photochemical as well as chemical [95] CXl methods are currently under research [2].

Photosynthetic pigments (chlorophylls and bacteriochlorophylls) that produce O_2_^−^ and ^•^OH radicals, with the consequent protein cross-linking, have been tested in rabbit cornea, giving a significant stiffening* in vivo* and* ex vivo*, after excitation with a near-infrared illumination [96]. The authors of this study found less adverse effects in the near-IR than after UVA exposure, but the toxicity levels of near-IR illumination on the eye need further evaluation [97].

In another study the Rose Bengal dye was excited by green light and led to an increased corneal stiffness, without toxicity to keratocytes [98].

Moreover* in vitro* studies suggest the possibility of substitution of UVA excitation of the riboflavin with one utilizing instead a femtosecond laser [12].

Using chemical CXL we can list various different methods that do not need any irradiance.

Most of the chemical cross-linkers however are toxic and cannot be used* in vivo* (e.g., formaldehyde and glutaraldehyde).

However to use *β*-nitroalcohols has been proposed, chemical molecules having a cross-linking mechanism similar to that of formaldehyde. Though they have never been tested for this purpose, we know from their numerous industrial applications that they have a positive safety record; for this reason they could be studied for use in CXL applications [99, 100]. Glyceraldehyde has been proven to function as a collagen cross-linker in a rabbit bullous keratopathy model [101].

Moreover a recent study also demonstrated that genipin (an active molecule derived from the plant* Gardenia jasminoides*) is also able to produce a CXL effect similar to CXL in porcine eyes, with minimal endothelial toxicity [102, 103].

## 9. Conclusions

CXL has revolutionized the management of these difficult progressive conditions such as KC and PMD, changing the clinical landscape from one of patiently watching a cornea degenerating without any ability to prevent the potential eventual requirement of a corneal graft. Instead there is a new arena whereby young people can be prevented from going down that intractable route of increasing morbidity and enabled instead to continue with their pastimes and other contact sports activities. It is therefore imperative that we continue to research this topic to ensure that this radical new therapy has long-term safety.

## Figures and Tables

**Table 1:** Possible complication after CXL.

Complication	Ref.	Notes	Treatment
Bacterial keratitis	[14–16]	Various organisms have been implicated with most commonly found to be of staphylococcal variant. The corneal epithelium is removed during the CXL treatment (epithelium-off method) to permit the diffusion of the riboflavin into the corneal stroma [17]. This step however reduces the immune-protective function of the superficial corneal layer against infectious agents [18].	Many surgeons are now forgoing the use of lenses postoperatively and increasing the frequency of antimicrobial drops to further reduce the risk of microbial infection after CXL [19]. Moreover linear abrasions can reduce the healing time and a CXL without removing the epithelium can be used [17, 18].
Acanthamoeba keratitis		Acanthamoeba keratitis is facilitated by the removal of the epithelium particularly if a CLenses is left in place.	

Herpes reactivation	[20]	It is well recognized that UV light can cause reactivation of herpes. This commonly occurs with those travelling to sunny climates or skiing in the winter.	Prophylactic systemic antiviral treatment in patients with history of herpetic disease.

Oedema	[21, 22]	Can be permanently caused by damage to the endothelial cells.	70% of CXL treated eyes show mild stromal oedema. Some significant cases were reported; however all of them resolved.

Haze	[23, 24]	In most of the cases it is temporary; only in 8-9% it was reported to last for long.	

Sterile infiltrates		UV treatment alters the response to antigens.	Reported in 7-8% of the cases, it can be treated with topical steroid.

Endothelial damage	[21, 22, 25, 26]	It happens in the case of a stromal thickness less than 400 *μ*m or incorrect focusing.	The threshold level of irradiance which could cause damage to the endothelium was found to be 0.35 mW/cm^2^, but this level is easily avoided if the corneal depth of 400 *μ*m is used as a cutoff level, with irradiance falling to 0.18 mW/cm^2^ when using the standard protocol. To date longer-term studies of corneal cross-linking have not shown any increased loss of endothelial cells after cross-linking compared to either the normal eyes or post-LASIK eyes [27, 28].

Treatment failure		7.6% of keratoconic progression following treatment at one-year followup [19].	—

**Table 2:** Devices used for CXL and their different features [2].

UV device	Procedure	Irradiance	Spot sizes
XLink*™* (Optos, Dunfermline, UK)	Standard 30 min CXL	0.5–5 mW/cm^2^	6, 8, and 10 mm

CBM Vega XLink Cross-Linking System (Carleton Optical, Chesham, UK)	Standard 30-min CXL	3 mW/cm^2^	4 to 11 mm

The LightLink CXL*™* (LightMed, San Clemente, Calif., USA)	From 3 to 30 min length protocols	Between 0.5 and 30 mW/cm^2^	4 to 11 mm

The UV-X*™* 2000 Cross-Linking System (IROC Innocross, Zurich, Switzerland)	Used for in a 10 min accelerated CXL procedure	12 mW/cm^2^	7.5 mm, 9.5 mm

KXL*™* System (Avedro, Waltham, Mass., USA)	Used in ultrafast accelerated CXL (<3 min of UVA exposure). It gave a positive outcome only in a small number of KC patients and in combination with the LASEK procedure	Intensity of 30 mW/cm^2^	Up to 11 mm
